# Bacterial nucleoid-associated protein HU as an extracellular player in host-pathogen interaction

**DOI:** 10.3389/fcimb.2022.999737

**Published:** 2022-08-23

**Authors:** Pavla Stojkova, Petra Spidlova

**Affiliations:** Department of Molecular Pathology and Biology, Faculty of Military Health Sciences, University of Defence, Hradec Kralove, Czechia

**Keywords:** HU protein, nucleoid-associated protein, histone-like protein, virulence, bacterial secretion, host-pathogen interaction

## Abstract

HU protein is a member of nucleoid-associated proteins (NAPs) and is an important regulator of bacterial virulence, pathogenesis and survival. NAPs are mainly DNA structuring proteins that influence several molecular processes by binding the DNA. HU´s indispensable role in DNA-related processes in bacteria was described. HU protein is a necessary bacterial transcription factor and is considered to be a virulence determinant as well. Less is known about its direct role in host-pathogen interactions. The latest studies suggest that HU protein may be secreted outside bacteria and be a part of the extracellular matrix. Moreover, HU protein can be internalized in a host cell after bacterial infection. Its role in the host cell is not well described and further studies are extremely needed. Existing results suggest the involvement of HU protein in host cell immune response modulation in bacterial favor, which can help pathogens resist host defense mechanisms. A better understanding of the HU protein’s role in the host cell will help to effective treatment development.

## Graphical Abstract

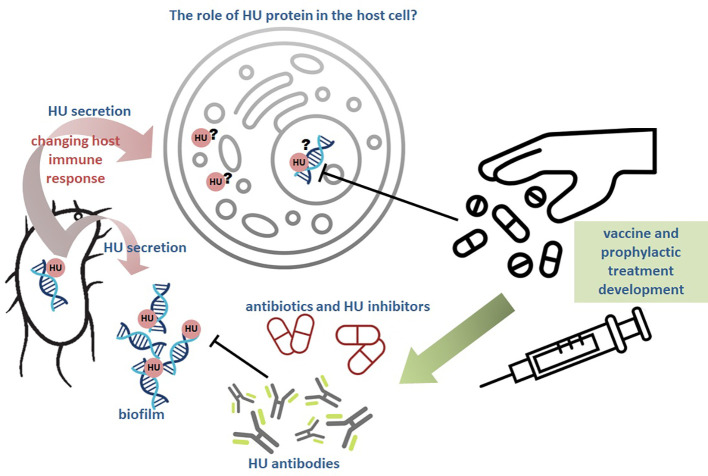

## Introduction

Nucleoid-associated proteins (NAPs), also called histone-like proteins (Pettijohn, 1988; Bahloul et al., 2001), are DNA structuring proteins that influence DNA compaction such as bridging, wrapping or bending (Swinger and Rice, 2007). These structural proteins are important regulators in bacteria and are necessary for bacterial virulence and pathogenesis (Stojkova et al., 2019). Among often discussed NAPs HU, IHF, H-NS, and FIS are listed (Dillon and Dorman, 2010). HU proteins exist, similarly to IHF homologue, as homo- or heterodimers. In most bacteria HU forms heterodimers consisting of two subunits (Pettijohn, 1988), but in some bacterial species it forms homodimers (Bhowmick et al., 2014; Oliveira Paiva et al., 2019; Stojkova et al., 2019). Roles of HU protein in many cellular processes, such as DNA compaction, shape modulation, replication, transcription, recombination (Broyles and Pettijohn, 1986; Roy et al., 2005; Oberto et al., 2009), negative supercoiling induction (Rouvière-Yaniv et al., 1979), regulation of the bacterial survival (Mangan et al., 2011; Priyadarshini et al., 2013; Ferrándiz et al., 2018; Stojkova et al., 2018), growth, SOS response (Preobrajenskaya et al., 1994; Oberto et al., 2009), virulence genes expression (Mangan et al., 2011; Stojkova et al., 2018), cell division (Oberto et al., 2009), and many others, were described. HU protein has been considered as an indispensable protein for many pathogenic bacteria, including Mycobacterium tuberculosis (Bhowmick et al., 2014), Francisella tularensis (Stojkova et al., 2018), Salmonella enterica (Mangan et al., 2011), Porphyromonas gingivalis (Priyadarshini et al., 2013), or Streptococcus pneumoniae (Ferrándiz et al., 2018). Given the importance of the HU protein in virulence and pathogenesis of many bacteria, HU protein could be a new target for therapeutics development. The role of the HU protein inside the bacterial cells is intensively studied, however its role in host-pathogen interaction is still largely unknown.

Secretion of bacterial proteins outside the cell and their delivery into the host cells is an important process in the world of pathogenic microorganisms. These virulence proteins can be crucial pieces in disease fighting and understanding the exact mechanisms of how they work and what is their key function could help suppress or treat the diseases.

### Secretion of the HU protein

HU protein was found to interact with bacterial lipopolysaccharide (LPS) (Thakur et al., 2021) suggesting HU protein is in close interaction with the outer membrane of Gram negative bacteria and may be released into extracellular space. In Gram positive bacteria the binding of the HU protein to another negatively charged molecule, the lipoteichoic acid, is suggested (Thakur et al., 2021). Not only HU protein binds LPS, but also extracellular DNA (eDNA) and thus it was suggested to act as a molecular glue (Thakur et al., 2021). The occurrence of HU protein in extracellular space was shown in M. tuberculosis (MDP1 protein, homologue of HU). Its role in promoting mycobacterial infection by mediating adhesion through the interaction with the host polysaccharide, the hyaluronic acid, was confirmed (Aoki et al., 2004). Interaction of HU protein with host protein was also shown in the case of Aggregatibacter actinomycetemcomitans, where HU protein interacts with host interleukine-1beta (IL-1β), suggesting HU protein could modulate pro-inflammatory response during infection (Paino et al., 2012). Moreover, antiserum against HU protein showed to be efficient in the reduction of bacteria and treatment of infection regarding peri-implantitis (Freire et al., 2017). HU protein was found to be secreted outside the bacterial cell into the host cell in the case of Wolbachia, where it was hypothesized that HU protein is delivered into the host cell nucleus because Wolbachia is localized in direct contact with nucleus and it is believed that bacterium secretes proteins through the nucleus membrane (Beckmann et al., 2013). Wolbachia disposes functional T4SS that allows secretion of proteins and DNA into extracellular space (Rancès et al., 2008). Recently, HU protein secretion through T4SS was described in Haemophilus (Jurcisek et al., 2017), thus direct Wolbachia HU protein secretion into host cell nucleus is presupposed. Due to the strong DNA binding properties of HU protein, it can be assumed that HU protein can modulate host genes expression. Likewise, HU protein is one of the secreted proteins by Helicobacter pylori into the culture supernatant, suggesting its contribution to gastric inflammation development (Kim et al., 2002). HU protein was detected in culture filtrate (Konecna et al., 2010) and extracellular vesicles (Klimentova et al., 2019) produced by F. tularensis, with an undescribed function yet. HU protein could also contribute to the virulence of Vibrio cholerae because HU protein is secreted into the medium and thus it could contribute to the cholera disease (Yan et al., 2022). Moreover, HU protein of V. cholerae is essential for CTXϕ phage replication that is responsible for virulence of V. cholerae (Martínez et al., 2015; Martínez et al., 2016). Streptococcus intermedius histone-like protein (Si-HLP), a HU protein homologue, is also released from the bacterium into the culture medium, as confirmed by electron microscopy (Liu et al., 2008). Moreover, recombinant Si-HLP stimulates the host immune system and activates the secretion of proinflammatory cytokines IL-8 and IL-1β, and tumor necrosis factor (TNF) through the extracellular signal-regulated kinase 1/2 (ERK1/2) and the c-Jun N-terminal kinase (JNK) pathways (Liu et al., 2008; Yumoto et al., 2019). Former studies showed that HlpA protein (Histone-like protein isolated from group A streptococci, HU homologue) has higher binding affinity to the glycosaminoglycans, such as heparin (Choi and Stinson, 1991; Winters et al., 1993) and that extracellular HlpA forms complexes with lipoteichoic acid *in vitro* (Stinson et al., 1998). HlpA of Streptococcus pyogenes is exposed on the surface (Severin et al., 2007) and is secreted into the medium (Lei et al., 2000). In addition, HlpA of Streptococcus mitis induces proinflammatory response in macrophages and thus contributes to the post-streptococcal glomerulonephritis (Zhang et al., 1999). The list of bacterial species secreting HU protein is shown in **Table 1**.

**Table 1:** List of bacterial species secreting HU protein (or HU homologue).

Bacteria	Extracellular localization	References
Escherichia coli	eDNA; LPS; biofilm	(Devaraj et al., 2015; Thakur et al., 2021)
Francisella tularensis	culture supernatant; vesicles	(Konecna et al., 2010; Klimentova et al., 2019)
Mycobacterium tuberculosis	extracellular; binds host polysaccharide	(Aoki et al., 2004)
Aggregatibacter actinomycetemcomitans	biofilm; interacts with host cytokine IL-1β	(Paino et al., 2012; Freire et al., 2017)
Wolbachia	host cell	(Beckmann et al., 2013)
Helicobacter pylori	culture supernatant	(Kim et al., 2002)
Vibrio cholera	culture supernatant	(Yan et al., 2022)
Streptococcus intermedius	culture supernatant	(Liu et al., 2008)
group A streptococci	interacts with heparin and lipoteichoic acid	(Choi and Stinson, 1991; Winters et al., 1993; Stinson et al., 1998)
Streptococcus pyogenes	surface-exposed; culture supernatant	(Lei et al., 2000; Severin et al., 2007)
Haemophilus influenzae	biofilm	(Brockson et al., 2014; Jurcisek et al., 2017)
Pseudomonas aeruginosa	biofilm	(Gustave et al., 2013; Novotny et al., 2016)
Burkholderia cenocepacia	biofilm	(Novotny et al., 2013)
Porphyromonas gingivalis	biofilm	(Rocco et al., 2017)
Streptococcus gordonii	biofilm	(Rocco et al., 2017)

The mechanisms of HU protein secretion are not fully described. One of the possible ways how HU protein is delivered through the bacterial membrane is by use of the T4SS secretion system, as described in Haemophilus influenzae, where HU protein is secreted outside the cell together with DNA in the early stages of attachment to the surface and becomes part of bacterial biofilm (Jurcisek et al., 2017). HU protein can be released also by other mechanism, including explosive lysis of the cells (Turnbull et al., 2016) or unknown secretion mechanisms. Mechanisms of HU protein secretion needs to be better investigated.

## HU protein as a part of the biofilm

Biofilm that is formed by many pathogenic bacteria contributes to the chronic infectious diseases and consists of natural polymers called extracellular polymeric substances (EPS), including extracellular DNA (eDNA), DNA-binding proteins (DNABII), pili, flagella, polysaccharides, and outer membrane vesicles (OMV) (Gunn, Bakaletz and Wozniak, 2016). Disruption of EPS may lead to better exposure of the pathogens and subsequently to their elimination using common antibiotics (Gunn et al., 2016). HU protein is a member of DNABII class of DNA-binding proteins and HU has been detected in bacterial biofilm in uropathogenic E. coli (UPEC) as well, and it has been shown that HU is a critical and limiting part of biofilm development and structural integrity of bacterial communities (Devaraj et al., 2015).

DNABII protein IHF, the homologue of HU, was found to be a target for the successful treatment of diseases caused by human pathogens, such as P. aeruginosa and H. influenzae, using monoclonal antibodies against IHF, where disruption of biofilms occurred (Brockson et al., 2014; Novotny et al., 2016). Moreover, IHF monoclonal antibodies treatment led to the eradication of Haemophilus and Pseudomonas biofilm *in vivo* (Novotny et al., 2016). Also in the case of oral infection, caused by A. actinomycetemcomitans, the hyper-immune antiserum targeting DNABII proteins was an effective treatment (Freire et al., 2017) and anti-IHF treatment has also been powerful against the chronic infections (Burkholderia cenocepacia, Pseudomonas aeruginosa) accompanying patients with cystic fibrosis (Gustave et al., 2013; Novotny et al., 2013). HU protein is a part of EPS of other human pathogens, Porphyromonas gingivalis and Streptococcus gordonii, and these biofilms can be disrupted by antisera targeting DNABII proteins, where DNABII proteins are limiting for S. gordonii but not for P. gingivalis biofilm formation (Rocco et al., 2017).

IHF protein shares structural and sequence similarity to the HU protein. Both proteins are widespread in prokaryotes. Due to their high level of homology, it can be assumed their comparable role. Moreover, some bacteria possess only single copy of DNABII, suggesting their promiscuity behavior. On the other hand, several differences in their specificity were found (Dey et al., 2017). The question arises, whether knowledge about IHF protein can indicate so far unexplored properties of the HU protein.

## Substances targeting HU protein

Several recent studies are focused on investigating the natural substances or chemicals targeting HU protein, which could inhibit its function or anyhow affect HU protein function on behalf of host immune defence. As we mentioned above, antibodies against DNABII (HU and IHF) proteins are effective substances that can abolish HU protein function and disrupt biofilm (Gustave et al., 2013; Novotny et al., 2013; Brockson et al., 2014; Novotny et al., 2016; Freire et al., 2017; Rocco et al., 2017), then bacteria are more sensitive to the commonly available antibiotics treatment (Gunn et al., 2016). Recently it was confirmed that the immuno-protective parts of IHF, a HU homologue, are inaccessible to the immune system when the proteins are bound to eDNA in biofilms and only non-protective C-terminal regions are exhibited. It was clearly demonstrated that antibodies against the immuno-protective region of IHF (DNA-binding tips) led to the biofilm collapse in H. influenzae whereas antibodies against IHF C- terminus were inefficient (Novotny et al., 2019). Due to the high homology of the HU protein to IHF similar functioning is expected. Our latest study suggests that arginine 58 (Arg58) of the HU protein in F. tularensis is necessary for DNA-binding capacity of the HU and bacterial strain with mutated Arg58 induces protective immune response *in vivo*, thus we assume that HU protein is not bound to the DNA and immuno-protective region of the protein is exposed to the immune system and effective antibodies are generated (Stojkova and Spidlova, under review).

Auspicious results showed a study by Zhang et al. (2022), where they investigated naturally occurring protein that acts as an HU protein inhibitor. Bacillus subtilis bacteriophage SPO1 protein Gp46 binds HU protein in its DNA-binding sites leading to the inhibition of HU protein function and overall bacterial (B. subtilis, M. tuberculosis, Acinetobacter baumannii, and Plasmodium falciparum) viability impairment. Because of the conservation of HU proteins across bacterial species, they suggest that the Gp46 protein could be a universal cross-species inhibitor of HU proteins and thus be an effective antimicrobial agent (Zhang et al., 2022).

Another effective antimicrobial treatment targeting HU protein is epigallocatechin gallate (EGCG). EGCG is a component of a green tea and its antimicrobial effect has been proven (Mabe et al., 1999; Taylor et al., 2005; Cui et al., 2012; Jeon et al., 2014; Nakayama et al., 2015; Lee et al., 2017; Parvez et al., 2019). A recent study on H. pylori showed that EGCG can bind HU protein and alter its function (Raj et al., 2021). Moreover, we have also confirmed the negative effect of EGCG on the function of the HU protein in F. tularensis which led to the overall attenuation of bacterial virulence both *in vitro* and *in vivo* (Stojkova et al., under review).

Effects of various chemical compounds on the HU protein function were studied. Among them, stilbene derivatives that inhibit HU protein binding to DNA in M. tuberculosis (Bhowmick et al., 2014) and HU homologue in African swine fever virus (ASFV) that lead to the decreased ability of replication in macrophages (Liu et al., 2020), bisphenol derivatives of fluorene (BDFs) that decrease DNA-binding capacity of the HU protein in mycoplasmas Spiroplasma melliferum and Mycoplasma gallisepticum; and E. coli (Agapova et al., 2020a; Agapova et al., 2020b). Other chemical compounds that could have negative effects on the stability of monomers of the S. melliferum HU protein and thus abolish the protein function were searched using virtual screening (Talyzina et al., 2017; Agapova et al., 2019; Agapova et al., 2020). Virtual screening was used also by a group of Dey and Ramakumar (2020), where they predict chemical substances that may have an inhibitory effect on mycobacterial HU protein, among them maltotetraose, valrubicin, iodixanol, enalkiren, indinavir, carfilzomib, oxytetracycline, quinalizarin, raltitrexed, epigallocatechin and their analogues.

## Future perspectives

HU protein is a small but very important protein for the survival and virulence of many pathogenic bacteria. Latest studies showed that this ordinary intracellular protein can be surface-exposed, be part of biofilm or can be delivered into the host cell after infection. Its function outside bacteria is not fully understood yet and further studies are needed. If the HU protein is specifically secreted into the host cell, its function at the site of localization needs to be elucidated. This multifunctional protein can perform many functions (not just DNA binding) that could affect the host cell response and these need to be explored. Small effector proteins, such as HU protein, can be a key in the fighting with dangerous diseases. Due to the wide distribution of HU proteins across bacterial species, it is quite likely that these proteins represent a universal therapeutic target for inhibitors and/or neutralizing antibodies.

## Author contributions

PaS and PeS - conceptualization, writing, review, editing. All authors contributed to the article and approved the submitted version.

## Funding

This work was supported by a Ministry of Defence of the Czech Republic - Long-term organization development plan Medical Aspects of Weapons of Mass Destruction of the Faculty of Military Health Sciences, University of Defence (DZRO-FVZ-ZHN-II).

## Conflict of interest

The authors declare that the research was conducted in the absence of any commercial or financial relationships that could be construed as a potential conflict of interest.

## Publisher’s note

All claims expressed in this article are solely those of the authors and do not necessarily represent those of their affiliated organizations, or those of the publisher, the editors and the reviewers. Any product that may be evaluated in this article, or claim that may be made by its manufacturer, is not guaranteed or endorsed by the publisher.
